# Subregional analysis of the amygdala, thalamus, and hypothalamus at the pre-decline stage in Parkinson’s disease with later cognitive impairment

**DOI:** 10.3389/fnagi.2025.1588027

**Published:** 2025-05-09

**Authors:** Kazuhide Seo, Genko Oyama, Toshimasa Yamamoto

**Affiliations:** ^1^Department of Neurology, Saitama Medical University, Saitama, Japan; ^2^Health Promotion Center, Saitama Medical University, Saitama, Japan

**Keywords:** Parkinson’s disease, cognitive decline, magnetic resonance imaging, brain structural analysis, amygdala, thalamus, hypothalamus

## Abstract

Cognitive decline in Parkinson’s disease (PD) significantly impacts patients’ quality of life, yet early detection remains challenging. While structural brain abnormalities in cortical regions have been widely documented using magnetic resonance imaging (MRI), subcortical regions have received less analytical attention despite their potential role as early biomarkers. This study investigated changes in specific subregions of the amygdala, thalamus, and hypothalamus in patients with PD before cognitive decline development. We analyzed MRI data from 163 participants (97 healthy controls [HC] and 66 patients with PD) from the Parkinson’s Progression Markers Initiative database. The patients with PD were classified based on cognitive status during a four-year follow-up: 21 who developed cognitive impairment (PDCI) and 45 who maintained normal cognition (PDNC). Cognitive function was assessed using the Montreal Cognitive Assessment and domain-specific tests. The PDCI group showed significantly lower corrected brain volumes in specific subregions of the amygdala (left basal nucleus), thalamus (bilateral lateral geniculate nuclei, right medial dorsal nucleus, and right anterior pulvinar nucleus), and hypothalamus (bilateral anterior-superior and left superior tubular parts) compared to that of HC. A significant difference between the PDCI and PDNC groups was observed only in the left lateral geniculate nucleus. In contrast, widespread structural changes were observed in cortical regions in the PDCI group, which showed stronger correlations with memory, attention, executive function, and visuospatial abilities. Hazard ratio analysis confirmed that structural changes in multiple cortical regions were significant predictors of cognitive decline. Although structural alterations were observed in subcortical regions, cortical changes demonstrated stronger associations with cognitive decline. These findings suggest that structural abnormalities may appear in the cerebral cortex before the stage proposed by conventional α-synuclein propagation models, potentially involving multiple mechanisms beyond α-synuclein, including global neural circuit dysfunction, disruption of neurotransmitter systems, breakdown of compensatory mechanisms, and coexisting pathologies (beta-amyloid and tau proteins). This study provides insights into early brain changes in PD and emphasizes the need for a comprehensive approach considering multiple mechanisms in early diagnosis and intervention strategies for PD-related cognitive impairment.

## 1 Introduction

Parkinson’s disease (PD) is a progressive neurodegenerative disorder characterized by both motor and non-motor symptoms (Kalia and Lang, 2015). Among the non-motor symptoms, cognitive impairment is particularly common and significantly impacts patients’ quality of life (Leroi et al., 2012). The average prevalence of mild cognitive impairment (MCI) in PD is 27% (range, 19–38%), and it is associated with an increased risk of developing dementia (Litvan et al., 2012). Cognitive decline in PD is multifactorial, involving various neurotransmitter systems and pathological processes, including dopaminergic, cholinergic, and noradrenergic deficits (Gratwicke et al., 2015), as well as the accumulation of alpha-synuclein and beta-amyloid proteins (Compta et al., 2011).

The Movement Disorder Society (MDS) has established diagnostic criteria for PD-MCI (Litvan et al., 2012), which include two levels of assessment: Level I criteria, which allow for a less comprehensive cognitive evaluation using global cognitive scales or a limited battery of neuropsychological tests, and Level II criteria, which require more detailed neuropsychological testing covering five cognitive domains. While both levels are validated diagnostic tools, they primarily rely on neuropsychological tests and may not capture the earliest stages of cognitive decline (Litvan et al., 2012). Although Level II criteria provide a more comprehensive assessment, the extensive testing required can be impractical in many clinical settings. Thus, more convenient and sensitive diagnostic tools are needed to detect cognitive impairment before it becomes clinically apparent. Early detection is crucial in planning appropriate care strategies and aids and in evaluating candidacy of invasive treatments such as deep brain stimulation.

Brain structural analysis using magnetic resonance imaging (MRI) has gained attention as a potential predictor for the onset of cognitive decline. Specifically, quantitative analyses of brain MRI have enabled the investigation of subtle changes in brain structure. Methods such as voxel-based morphometry, cortical thickness analysis, and diffusion tensor imaging (DTI) allow for quantitative assessment of gray matter volume, cortical thickness, and white matter microstructure (Lerch et al., 2017). These techniques can detect early brain structural changes that are difficult to identify through conventional visual assessment, aiding in the early detection and prediction of cognitive impairment progression (Lanskey et al., 2018).

Multiple meta-analyses have revealed structural changes associated with cognitive decline in PD, particularly, gray matter atrophy in PD-MCI affecting the frontal, temporal, and parietal lobes, as well as the insula and limbic system (Hou and Shang, 2022; Zheng et al., 2019; Mihaescu et al., 2019; Xu et al., 2016). Furthermore, studies focusing on PD-MCI converters have shown cortical thinning at baseline in the anterior cingulate, parietal, temporal, and occipital cortices. These studies have also documented atrophy in the nucleus accumbens and progressive volume loss in the thalamus, caudate nucleus, nucleus accumbens, and hippocampal CA2–3 regions (Mak et al., 2015; Foo et al., 2017b; Gorges et al., 2020; Filippi et al., 2020; Foo et al., 2017a). These findings suggest that structural changes in the brain beginning in the cognitively normal stage are associated with subsequent transition to MCI.

While many studies have reported structural abnormalities primarily in cortical regions, subcortical regions have not been analyzed in much detail as cortical regions. Recent advances in MRI analysis techniques have enabled the analysis of fine subregions in areas such as the hippocampus (Iglesias et al., 2015), amygdala (Saygin et al., 2017), thalamus (Iglesias et al., 2018), and hypothalamus (Billot et al., 2020). This provides an opportunity to capture subtle structural changes in these regions and better understand the mechanisms of cognitive decline in PD. Several studies have conducted detailed subregional analyses of the hippocampus. For instance, patients with PD-MCI have shown volume reductions in CA1 and the hippocampal–amygdaloid transition area (HATA) compared with healthy controls (HCs) and patients with PD-normal cognitive impairment (NCI) (Becker et al., 2021). Moreover, the volumes of hippocampal subregions, particularly the hippocampal para-gyrus, CA4, granule cell layer, and HATA regions, have been shown to be important predictors of transition from PD-NCI to PD-MCI (Foo et al., 2017b; Kandiah et al., 2014b).

However, few studies have conducted detailed analyses of the subregions of the amygdala, thalamus, and hypothalamus to investigate structural abnormalities prior to MCI transition. These subcortical structures may be affected by α-synuclein propagation earlier than cortical areas and are involved in various cognitive processes. The amygdala shows accumulation of α-synuclein pathology from early stages, leading to dysfunction in emotional and memory processing (Braak et al., 1994; Huang et al., 2015). Impairment of cholinergic neurotransmission in the thalamus is thought to play a crucial role in the onset and progression of cognitive symptoms in PD (Ziabreva et al., 2006). The hypothalamus in PD exhibits widespread α-synuclein pathology (Sandyk et al., 1987), resulting in reduced functional connectivity with other brain regions. This pathological change leads to dysregulation of the autonomic nervous system, affecting blood pressure control and thermoregulation (Dayan et al., 2018). Such autonomic dysfunction may indirectly impact cognitive function, possibly through changes in cerebral hemodynamics (Kim et al., 2012). Thus, the predictive value of subregions within the amygdala, thalamus, and hypothalamus for PD-MCI has not yet been elucidated. Our research focuses on the potential of these subcortical structures (the amygdala, thalamus, and hypothalamus) as early biomarkers for predicting cognitive decline in PD. The main focus of this study is to leverage recent advances in MRI analysis techniques to examine fine subregions of the amygdala, thalamus, and hypothalamus—structures that have been technically challenging to analyze in detail until now. We aim to investigate whether structural abnormalities in these specific subregions exist before PD transitions to MCI. This comprehensive approach may enable us to capture more precise patterns of brain structural changes from the early stages of cognitive decline in PD, potentially contributing to its early diagnosis and prognosis.

## 2 Materials and methods

### 2.1 Research participants

All MRI and clinical data used in this study were obtained from the Parkinson’s Progression Markers Initiative (PPMI) database.^1^ According to the PPMI cohort criteria, patients with PD had to meet the following conditions at baseline: (i) have at least two of the following: resting tremor, bradykinesia, rigidity, or either asymmetric resting tremor or asymmetric bradykinesia; (ii) be in an early clinical stage (within 2 years of PD diagnosis and have Hoehn and Yahr [H&Y] stage I or II); (iii) not be taking PD medication; (iv) have confirmed dopamine transporter deficiency on imaging; (v) be judged as not having dementia by the research site investigator; (vi) have a 15-item Geriatric Depression Scale (GDS-15) score < 5. The HC group met the following criteria: (i) no significant neurological dysfunction and (ii) no first-degree relatives with PD.

From the PPMI cohort, we selected participants meeting our MRI criteria and further excluded those with (i) poor image quality, (ii) history of cerebrovascular disease or head trauma that could affect brain image analysis, (iii) baseline Montreal Cognitive Assessment (MoCA) score < 26, or (iv) missing baseline clinical data. The final sample consisted of 163 participants: 97 HC and 66 patients with PD.

**Figure 1:**
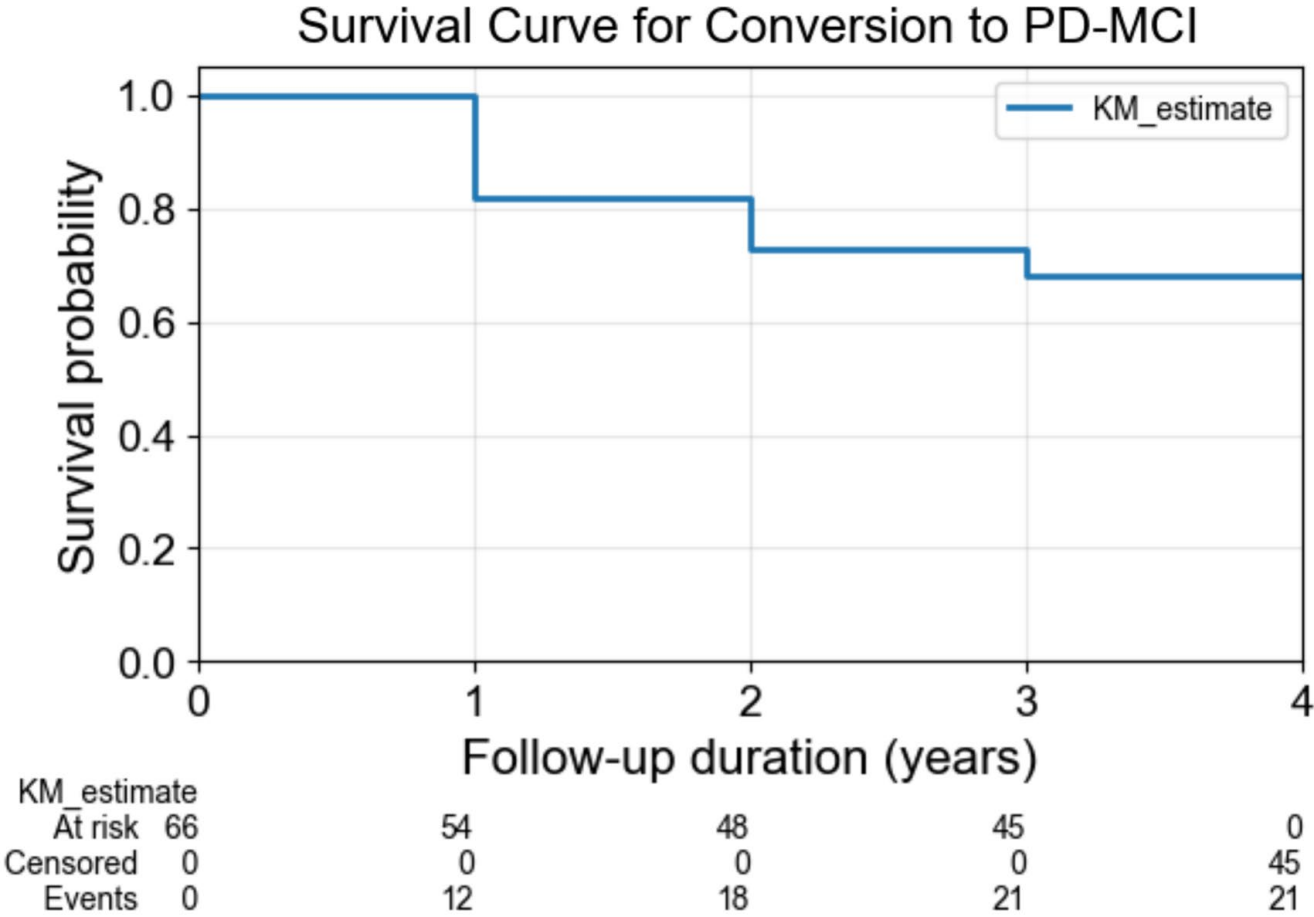
Survival curve for conversion to PD-MCI. The Kaplan–Meier survival curve illustrates the cumulative number of cognitive impairment cases among patients with PD over the 4-year follow-up period. PD-MCI: Parkinson’s Disease–Mild Cognitive Impairment.

Patients with PD were followed up annually during a 4-year follow-up period. We divided patients with PD into two groups based on their cognitive status at each annual assessment: 21 patients with PD who showed cognitive impairment (PDCI) and 45 patients with PD who maintained normal cognitive function (PDNC). For the cognitive assessment, we utilized MoCA, which has been widely established as a validated assessment tool for cognitive function in PD even before the publication of the MDS Task Force guidelines (Dalrymple-Alford et al., 2010). While Level II diagnosis in the guidelines enables comprehensive evaluation using multiple tests across five cognitive domains (attention/working memory, memory, language, executive function, and visuospatial function), such extensive neuropsychological testing is often impractical in clinical settings owing to time constraints. Studies have demonstrated that Level I criteria, including MoCA, show comparable effectiveness to Level II criteria in predicting transition to PD-MCI (Hoogland et al., 2019), and MoCA specifically has proven reliable in predicting long-term cognitive outcomes (Kandiah et al., 2014a). Therefore, PDCI was defined as participants whose MoCA score fell below 26 during the follow-up period, following Level I of the MDS PD-MCI diagnostic criteria (Litvan et al., 2012). This approach aligns with current clinical practice and has been widely adopted in predictive modeling studies of cognitive decline in PD (Schrag et al., 2017). The Kaplan–Meier survival curve in Figure 1 illustrates the cumulative number of cognitive impairment cases among patients with PD over the 4-year follow-up period.

### 2.2 Study design

This study retrospectively analyzed data from the PPMI cohort and adopted a longitudinal design. In PPMI study, all participants underwent baseline MRI scans and clinical evaluations. Patients received annual follow-up examinations for 4 years, with repeated cognitive function assessments using MoCA.

### 2.3 Magnetic resonance imaging

All MRI examinations were conducted using a 3.0 T MRI scanner (Siemens Healthcare, USA) following a standard protocol. A 3D magnetization-prepared rapid gradient-echo sequence was used for brain anatomy imaging (176 axial slices, repetition time = 2,300 ms, echo time = 2.98 ms, flip angle = 9°, voxel size 1 mm × 1 mm × 1 mm). A gradient-echo echo-planar imaging sequence over 210 volumes or time points was used for resting-state brain functional activity imaging (40 axial slices, repetition time = 2,400 ms, echo time = 25 ms, flip angle = 80°, voxel size 3.3 mm × 3.3 mm × 3.3 mm). Detailed information can be found at https://www.ppmi-info.org/study-design/researchdocuments-and-sops/.

### 2.4 Image analysis

T1-weighted images were processed using FreeSurfer (version 7.3.2). The processing pipeline is as follows: Firstly, image preprocessing (skull stripping, intensity normalization, white matter and gray matter classification, etc.) is performed. Subsequently, each brain region is automatically labeled, and quantitative metrics such as volume, thickness, and surface area are calculated for each anatomically defined region of interest. FreeSurfer is available for free download and use online.^2^

The thalamus, amygdala, hippocampus, brainstem, and hypothalamus were analyzed using the Subregion Segmentation tool.^3^^,^^4^ The brain was segmented into 68 cortical, 16 deep gray matter, 70 white matter, 4 cerebellar, 38 hippocampal, 18 amygdala, 50 thalamic regions, 10 hypothalamic, and 4 brainstem regions.

The hippocampus, amygdala, thalamus, and hypothalamus were divided into the following regions for both left and right hemispheres:

Hippocampus (19 regions): cornu ammonis 1-body (CA1-body), CA1-head, CA3-body, CA3-head, CA4-body, CA4-head, fimbria, granule cells-molecular layer-dentate gyrus-body (GC-ML-DG-body), GC-ML-DG-head, HATA, hippocampal tail, hippocampal fissure, molecular layer-hippocampus-body (molecular-layer-HP-body), molecular-layer-HP-head, parasubiculum, presubiculum-body, presubiculum-head, subiculum-body, and subiculum-head.

Amygdala (9 regions): accessory basal nucleus, anterior amygdaloid area, basal nucleus, central nucleus, cortical nucleus, corticoamygdaloid transition area, lateral nucleus, medial nucleus, and paralaminar nucleus.

Thalamus (25 regions): anterior ventral nucleus, central medial nucleus, central lateral nucleus, centromedian nucleus, lateral part of the suprageniculate nucleus, lateral dorsal nucleus, lateral geniculate nucleus, lateral posterior nucleus, medial dorsal nucleus lateral part, medial dorsal nucleus medial part, medial geniculate nucleus, nucleus reuniens, paracentral nucleus, parafascicular nucleus, paratenial nucleus, anterior pulvinar nucleus, inferior pulvinar nucleus, lateral pulvinar nucleus, medial pulvinar nucleus, ventral anterior nucleus, ventral anterior nucleus magnocellular part, ventral lateral nucleus anterior part, ventral lateral nucleus posterior part, ventral medial nucleus, and ventral posterolateral nucleus.

Hypothalamus (5 regions): anterior-inferior part (suprachiasmatic nucleus, supraoptic nucleus), anterior-superior part (preoptic area, paraventricular nucleus), posterior part (mamillary body, lateral hypothalamus, tuberomamillary nucleus), inferior tubular part (infundibular nucleus, ventromedial nucleus, supraoptic nucleus, lateral tubular nucleus), and superior tubular part (dorsomedial nucleus, paraventricular nucleus, lateral hypothalamus).

This detailed segmentation allowed for the assessment of subtle structural changes in each brain region.

Clinical and neuropsychological assessments disease stage was evaluated using the H&Y scale, and motor symptoms were assessed using the MDS–Unified Parkinson’s Disease Rating Scale (MDS-UPDRS). Depressive symptoms were evaluated using GDS-15. Cognitive function was assessed using MoCA and domain-specific tests. Domain-specific tests included the Hopkins Verbal Learning Test-Revised for memory, Benton Judgment of Line Orientation 15-item (half) version for visuospatial function, Symbol-Digit Modalities Test (SDMT) for processing speed and attention, and Letter-Number Sequencing (LNS) and semantic fluency (animals) for executive function and working memory.

### 2.5 Comparison of corrected brain volumes

Corrected brain volumes were calculated by dividing measured volumes by intracranial volume. Group comparisons were conducted using analysis of covariance (ANCOVA), adjusting for age, sex, handedness, years of education, and GDS-15 scores as covariates. As this was an exploratory analysis, multiple comparisons were corrected using the least significant difference (LSD) method.

### 2.6 Association between cognitive test scores and brain regions

Associations between corrected volumes of brain regions that showed significant differences between the PDCI and PDNC groups were examined using ANCOVA. Age, sex, handedness, years of education, and GDS-15 scores were included as covariates. As this was an exploratory analysis, multiple comparisons were corrected using the LSD method.

### 2.7 Hazard ratios for MCI progression

For brain regions that showed significant differences between the PDCI and PDNC groups, Z-scores of corrected brain volumes were calculated using the entire PD sample as the reference population. Based on these Z-scores, survival analysis using the Cox proportional hazards model was performed to estimate hazard ratios for progression to MCI. The event was defined as the point at which the MoCA score fell below 26. The model was adjusted for age, sex, handedness, years of education, and GDS-15 scores. To correct for multiple comparisons, the false discovery rate (FDR) method was applied.

### 2.8 Demographic and clinical characteristics

Continuous variables such as age, years of education, GDS scores, and neuropsychological test scores were compared between groups using the Mann–Whitney U test. Categorical variables such as sex and handedness were analyzed using the chi-squared test. Multiple comparisons were adjusted using the LSD method.

### 2.9 Statistical analysis

All statistical analyses were performed using Python 3.13.1^5^ on Windows via the command-line interface.

The following libraries were used with their latest available versions at the time of analysis:

- pandas (v2.2.1) for data handling,
- statsmodels (v0.14.1) for ANCOVA and multiple comparisons using both the LSD method and the FDR method,
- lifelines (v0.27.8) for Kaplan–Meier survival analysis and Cox proportional hazards modeling,
- scipy.stats (v1.13.0) for Mann–Whitney U test and chi-squared tests for categorical variables,
- matplotlib (v3.8.3) for data visualization.

All analyses were conducted using custom scripts written by the authors. All packages were freely available from their respective official sources:

- pandas: https://pandas.pydata.org
- statsmodels: https://www.statsmodels.org
- lifelines: https://lifelines.readthedocs.io
- scipy: https://scipy.org
- matplotlib: https://matplotlib.org

## 3 Results

### 3.1 Demographic and clinical characteristics

As shown in Table 1, the PDCI group showed significantly lower scores than the HC and PDNC groups in HVLT-R immediate recall, delayed recall, and SDMTOTAL. Additionally, the PDNC group showed significantly lower scores than the HC groups in HVLT-R immediate recall.

**Table 1:** Demographic and clinical characteristics of study participants.

	HC (*n* = 97)	PDNC (*n* = 45)	PDCI (*n* = 21)	*p*-value		
				HC vs. PDNC	HC vs. PDCI	PDNC vs. PDCI
Age (year)	60 (10.9)	57.8 (9.8)	62.6 (8)	0.4228	0.7101	0.1889
Sex (m/f)	60 (37)	25 (20)	18 (3)	0.5970	0.1973	0.1973
Handed (right/left/mixed)	81/7/9	38/6/1	20/0/1	0.2888	0.3977	0.2888
Education (year)	15.7 (3.0)	15.2 (2.6)	15.7 (3.0)	0.699	0.8777	0.8434
Duration (year)	–	2.4 (3.1)	1.8 (1.2)	–	–	0.8434
Hoehn and Yahr stage (I/II)	–	24/21	10/11	–	–	0.8434
MDS-UPDRS Part III	–	18.1 (7.1)	24 (9.7)	–	–	0.1040
GDS	5.2 (1.5)	5.2 (1.4)	5.2 (1.5)	0.8014	0.8014	0.8777
MoCa	28.3 (1.2)	28.4 (1.3)	27.8 (1.2)	0.8500	0.1499	0.1390
JoLO	13.4 (1.7)	13.2 (1.9)	13.1 (2.0)	0.8777	0.8777	0.9548
HVLT-R immediate recall	25.9 (4.4)	27.8 (4.6)	22.9 (4.0)	0.0483*	0.0441*	0.0014*
HVLT-R delayed recall	9.2 (2.2)	10 (1.6)	7.5 (2.7)	0.2152	0.0441*	0.0014*
LNS	11.4 (2.6)	11.7 (1.6)	10 (2.2)	0.8014	0.1499	0.1096
SDMTOTAL	48 (10.5)	43.9 (7.6)	37.4 (8.4)	0.0830	0.0014*	0.0441*
Semantic fluency total (animal)	22.6 (5.1)	21.8 (4.9)	20.5 (4.9)	0.6801	0.3771	0.8014

HC, Healthy control; PDNC, Parkinson’s disease with normal cognition; PDCI, Parkinson’s disease with cognitive impairment; MDS-UPDRS, Movement Disorder Society–Unified Parkinson’s Disease Rating Scale; GDS, Geriatric Depression Scale; MoCA, Montreal Cognitive Assessment; JoLO, Judgment of Line Orientation; HVLT-R, Hopkins Verbal Learning Test-Revised; LNS, Letter-Number Sequencing; SDMT, Symbol Digit Modalities Test. *p < 0.050.

### 3.2 Comparison of corrected brain volumes

Significant differences were observed in specific subregions of the amygdala, thalamus, hypothalamus, and hippocampus (Table 2). In the amygdala, the PDCI group showed significantly lower corrected brain volumes in the left basal nucleus than the HC group. In the thalamus, the PDCI group showed significantly lower corrected brain volumes in the bilateral lateral geniculate nuclei, right medial dorsal nucleus medial part, and right anterior pulvinar nucleus than that of the HC group. Furthermore, the PDCI group showed significantly lower corrected brain volumes in the left lateral geniculate nucleus than that of the PDNC group. The PDNC group showed significantly lower corrected brain volumes in the right medial dorsal nucleus medial part, and medial dorsal nucleus lateral part than that of the HC group. In the hypothalamus, the PDCI group showed significantly lower corrected brain volumes in the bilateral anterior-superior part, left superior tubular part, and left total than that of the HC group.

**Table 2:** Between-group comparison of corrected brain volumes.

Brain area		HC > PDNC		HC < PDNC		HC > PDCI		HC < PDCI		PDNC > PDCI		PDNC < PDCI	
		Anatomical regions	*p*-value	Anatomical regions	*p*-value	Anatomical regions	*p*-value	Anatomical regions	*p*-value	Anatomical regions	*p*-value	Anatomical regions	*p*-value
Cortical	Frontal lobe	–		–		–		rh-caudal anterior cingulate cortex	0.0372	–		lh-rostral anterior cingulate	0.0330
	Temporal lobe	–		lh-transverse temporal gyrus	0.0224	rh-superior temporal gyrus	0.0382	–		rh-superior temporal gyrus	0.0109	–	
						rh-middle temporal gyrus	0.0191			lh-superior temporal gyrus	0.0081		
										rh-middle temporal gyrus	0.0148		
										lh-middle temporal gyrus	0.0494		
	Parietal lobe	–		rh-inferior parietal lobule	0.0485	–		–		rh-inferior parietal lobule	0.0174	–	
	Occipital lobe	–		–		–		–		rh-lateral occipital gyrus	0.0197	–	
Subcortical gray matter		–		–		rh-caudate nucleus	0.0225	–		rh-putamen	0.0278	–	
						lh-caudate nucleus	0.0127			rh-caudate	0.0495		
White matter		WM of lh-pars triangularis	0.0405	–		WM of rh-frontal pole	0.0249	–		WM of lh-transverse temporal gyrus	0.0436	–	
		WM of rh-parahippocampal gyrus	0.0395			WM of lh-precentral gyrus	0.0237						
		WM of rh-fusiform gyrus	0.0039			WM of rh-lateral occipital gyrus	0.0314						
		WM of rh-pericalcarine gyrus	0.0220			WM of lh-lateral occipital gyrus	0.0425						
		WM of rh-lingual gyrus	0.0027			WM of rh-supramarginal gyrus	0.0377						
		central-corpus callosum	0.0443										
Hippocampus		–		–		lh-paralaminar nucleus	0.0113	–		–		–	
Amygdala		–		–		lh-basal nucleus	0.0438	–		–		–	
Thalamic		rh-medial dorsal nucleus medial part	0.0475	–		rh-lateral geniculate nucleus	0.0398	–		lh-lateral geniculate nucleus	0.0255	–	
		rh-medial dorsal nucleus lateral part	0.0384			lh-lateral geniculate nucleus	0.0107						
						rh-medial dorsal nucleus medial part	0.0461						
						anterior pulvinar nucleus	0.0415						
Hypothalamic subunits		–		–		rh-anterior-superior part	0.0406	–		–		–	
						lh-anterior-superior part	0.0243						
						lh-superior tubular part	0.0109						
						whole-left	0.0214						

HC, Healthy control; PDNC, Parkinson’s disease with normal cognition; PDCI, Parkinson’s disease with cognitive impairment. *p < 0.050.

In the hippocampal subregions, the PDCI group showed significantly lower corrected brain volumes in the left paralaminar nucleus than that of the HC group.

Significant differences were also observed in other brain regions. The PDCI group showed significantly lower corrected brain volumes than that of the HC group in temporal lobe (right superior temporal gyrus and right middle temporal gyrus), subcortical gray matter (the bilateral caudate nuclei), and white matter regions (right frontal pole, left precentral gyrus, the bilateral lateral occipital gyri, and right supramarginal gyrus). Conversely, the PDCI group showed significantly higher corrected brain volumes in the right caudal anterior cingulate cortex than that of the HC group.

The PDCI group showed significantly lower corrected brain volumes than that of the PDNC group in the temporal lobe (the bilateral superior temporal gyri and bilateral middle temporal gyri), parietal lobe (right inferior parietal lobule), occipital lobe (right lateral occipital gyrus), subcortical gray matter (right putamen and right caudate nucleus), and white matter regions (left transverse temporal). Conversely, the PDCI group showed significantly higher corrected brain volumes in the left rostral anterior cingulate than that of the PDNC group.

The PDNC group showed significantly lower corrected brain volumes than that of the HC group in the white matter regions (left pars triangularis, right parahippocampal gyrus, right fusiform gyrus, right pericalcarine gyrus, right lingual gyrus, and central-corpus callosum). However, the PDNC group showed significantly higher corrected brain volumes in the left transverse temporal gyrus and right inferior parietal lobule than that of the HC group.

### 3.3 Association between cognitive test scores and brain regions

We analyzed the association between cognitive test scores and brain regions that showed significant differences in corrected brain volumes between the PDCI and PDNC groups (Table 3).

**Table 3:** Association of corrected brain volumes with cognitive test scores.

Brain area		Anatomical regions	V	M		AW		E	MoCA						
			JoLO	HVLT-R immediate recall	HVLT-R delayed recall	LNS	SDMTOTAL	Semantic fluency total (animal)	VISUOSPATIAL / EXECUTIVE	NAMING	ATTENTION	LANGUAGE	DELAYED RECALL	ORIENTATION	TOTAL
			correlation coefficient (*p*-value)												
Cortical	Frontal lobe	lh-rostralanteriorcingulate	0.2604 (0.0347*)	−0.0601 (0.6314)	−0.1651 (0.1853)	−0.0034 (0.9783)	0.0076 (0.9518)	−0.0202 (0.8721)	−0.1113 (0.3737)	0.0498 (0.6915)	−0.2619 (0.0337*)	−0.0459 (0.7146)	−0.1651 (0.1853)	0.134 (0.2835)	−0.0025 (0.9844)
	Temporal lobe	rh-middletemporal	0.0948 (0.4491)	0.155 (0.214)	0.1061 (0.3966)	−0.0205 (0.8703)	0.145 (0.2454)	−0.0773 (0.5375)	0.1716 (0.1684)	0.0351 (0.7795)	−0.0378 (0.7633)	−0.0778 (0.5347)	0.1061 (0.3966)	0.1362 (0.2754)	0.2218 (0.0735)
		lh-middle temporal gyrus	0.0423 (0.7361)	0.2073 (0.0948)	0.0786 (0.5307)	−0.0641 (0.6091)	0.0689 (0.5828)	0.0308 (0.8058)	0.196 (0.1148)	−0.0974 (0.4365)	−0.0809 (0.5185)	−0.0817 (0.5145)	0.0786 (0.5307)	0.0512 (0.6828)	0.1945 (0.1176)
		rh-superiortemporal	0.0582 (0.6425)	0.1134 (0.3645)	0.1128 (0.3672)	−0.0767 (0.5405)	0.0463 (0.712)	−0.0399 (0.7507)	0.0888 (0.4781)	0.1588 (0.2027)	−0.1187 (0.3423)	−0.148 (0.2357)	0.1128 (0.3672)	0.2192 (0.077)	0.1538 (0.2175)
		lh-superiortemporal	0.1075 (0.3901)	0.1974 (0.1122)	−0.0907 (0.469)	0.0156 (0.9012)	0.279 (0.0233*)	0.0657 (0.6003)	0.0714 (0.5691)	0.0425 (0.7346)	0.0628 (0.6164)	−0.0204 (0.871)	−0.0907 (0.469)	0.1924 (0.1217)	0.2838 (0.0209*)
	Parietal lobe	rh-inferiorparietal	0.1138 (0.3628)	−0.0092 (0.9418)	0.0346 (0.7825)	0.1742 (0.1618)	0.136 (0.2762)	−0.0631 (0.6145)	0.2735 (0.0263*)	0.1204 (0.3356)	−0.0055 (0.9649)	−0.0499 (0.6905)	0.0346 (0.7825)	0.1422 (0.2547)	0.2127 (0.0865)
	Occipital lobe	rh-lateraloccipital	0.028 (0.8236)	0.124 (0.3213)	0.0801 (0.5226)	0.1906 (0.1253)	0.1699 (0.1727)	0.1202 (0.3364)	0.1771 (0.1548)	0.0558 (0.6563)	−0.0646 (0.6061)	0.0366 (0.7706)	0.0801 (0.5226)	0.2019 (0.104)	0.2838 (0.0209)
Subcortical gray matter		rh-putamen	0.0338 (0.7876)	−0.0591 (0.6373)	0.1008 (0.4206)	−0.0158 (0.9)	0.185 (0.1369)	−0.1342 (0.2828)	0.2183 (0.0783)	0.1301 (0.2977)	−0.023 (0.8544)	0.0851 (0.4968)	0.1008 (0.4206)	−0.1147 (0.3592)	−0.0764 (0.5421)
		rh-caudate	−0.1158 (0.3544)	0.0658 (0.5998)	−0.0704 (0.5745)	−0.0271 (0.829)	0.1773 (0.1545)	0.0203 (0.8714)	0.0203 (0.8714)	0.0123 (0.9217)	0.0091 (0.9421)	−0.0271 (0.829)	−0.0704 (0.5745)	0.0123 (0.9217)	−0.0311 (0.8043)
White matter		wm-lh-transversetemporal	−0.2529 (0.0404*)	0.2342 (0.0584)	0.039 (0.756)	0.0225 (0.8574)	0.0647 (0.6059)	−0.0524 (0.6758)	0.1021 (0.4146)	−0.1179 (0.3457)	−0.0263 (0.8341)	−0.0038 (0.976)	0.039 (0.756)	−0.1047 (0.403)	0.1369 (0.2731)
Thalamic		lh-lateral-geniculate	−0.0339 (0.7873)	0.1597 (0.2001)	0.2068 (0.0957)	0.1479 (0.236)	0.0388 (0.757)	−0.2043 (0.0999)	0.237 (0.0553)	−0.1469 (0.2392)	−0.0102 (0.9351)	−0.0867 (0.4891)	0.2068 (0.0957)	−0.1453 (0.2443)	0.0388 (0.757)

PDNC, Parkinson’s disease with normal cognition; PDCI, Parkinson’s disease with cognitive impairment; JoLO, Judgment of Line Orientation; HVLT-R, Hopkins Verbal Learning Test-Revised; LNS, Letter-Number Sequencing; SDMTOTAL, Total score of Symbol Digit Modalities Test; MoCA, Montreal Cognitive Assessment. *p < 0.050.

Positive correlations were observed between regional brain volumes and cognitive performance measures. Specifically, the left superior temporal gyrus was significantly associated with SDMTOTAL, the right lateral occipital gyrus with HVLT-R immediate recall, the right inferior parietal lobule with the visuospatial/executive function component of MoCA, and the left rostral anterior cingulate with JoLO score. Conversely, negative correlations were found between the left rostral anterior cingulate and the attention function component of MoCA, as well as between the white matter of left transverse temporal gyrus and JoLO score.

### 3.4 Hazard ratios for MCI progression

Table 4 presents the hazard ratios for MCI progression calculated based on the corrected brain volumes of portions of brain regions that showed significant differences between the PDCI and PDNC groups. Significant hazard ratios were observed in the bilateral rostral anterior cingulate, superior temporal gyri, middle temporal gyri, right inferior parietal lobule, right lateral occipital gyrus, white matter of the left transverse temporal gyrus, and the left lateral geniculate nucleus, indicating that reduced corrected brain volumes in these regions were associated with an increased risk of cognitive decline.

**Table 4:** Hazard ratios for MCI progression in brain regions with significant differences between PDCI and PDNC.

Anatomical regions	Hazard ratio per 1 SD	*p*-value
lh-rostral anterior cingulate	1.6600	0.0365*
rh-superior temporal gyrus	2.5689	0.0220*
lh-superior temporal gyrus	2.4047	0.0220*
rh-middle temporal gyrus	2.7578	0.0220*
lh-middle temporal gyrus	2.1082	0.0365*
rh-inferior parietal lobule	1.6099	0.0365*
rh-lateral occipital gyrus	1.5418	0.0456*
rh-putamen	1.6971	0.0713*
rh-caudate	1.3833	0.1939
WM of lh-transverse temporal gyrus	1.6676	0.0417*
lh-lateral geniculate nucleus	2.0850	0.0224*

MCI, mild cognitive impairment; PDNC, Parkinson’s disease with normal cognition; PDCI, Parkinson’s disease with cognitive impairment. *p < 0.050.

## 4 Discussion

This study provides important insights into early structural brain changes that occur prior to the onset of cognitive impairment in patients with PD through detailed subregional analysis of subcortical structures. Our analysis revealed specific structural alterations in the amygdala (left basal nucleus), thalamus (the bilateral lateral geniculate nuclei, right medial dorsal nucleus medial part, and right anterior pulvinar nucleus), and hypothalamus (the bilateral anterior-superior part, left superior tubular part, and left total) in patients with PDCI compared with the HC. In addition, PDNC patients exhibited significant structural changes in the thalamus (the right medial dorsal nucleus medial part and medial dorsal nucleus lateral part) relative to those observed in HC. These changes have not been previously reported at this level of anatomical detail.

In PD, the spread of alpha-synuclein pathology has been classically explained by the staged pattern proposed by Braak et al. (2003). This model describes how abnormal alpha-synuclein (Lewy body) pathology begins in the lower brainstem and gradually ascends through the limbic system to the cerebral neocortex. Specifically, lesions first appear in the dorsal nucleus of the vagus nerve in the medulla and the olfactory bulb (Stage 1), then progress to the pontine reticular formation and locus coeruleus (Stage 2), the substantia nigra and amygdala (Stage 3), followed by the limbic cortex and thalamus (Stage 4), the higher association areas of the neocortex (Stage 5), and finally the primary motor and sensory cortices (Stage 6). The “dual-hit hypothesis” (Hawkes et al., 2007) complements this progression model by suggesting that the pathogenic agent (likely a virus or prion-like factor) enters the central nervous system through both nasal and gastrointestinal mucosa. Specifically, one pathway leads from the olfactory mucosa to the olfactory bulb and then to the limbic system of the temporal lobe, while the other pathway travels from the enteric plexus, retrograde along the vagus nerve to the medullary vagal nucleus, and then ascends to reach the substantia nigra. As an extension of this dual-hit hypothesis, recent research proposes two PD subtypes: “Body-first” and “brain-first” (Borghammer and Van Den Berge, 2019; Borghammer, 2021). In the body-first type, pathology begins in peripheral systems such as the enteric nervous system and ascends via the vagus nerve or sympathetic ganglia to the lower brainstem, then progresses upward to the substantia nigra, limbic system, and cerebral cortex, following a course similar to the Braak model. This type tends to present with autonomic dysfunction and REM sleep behavior disorder before motor symptoms appear. In contrast, the brain-first type begins within the central nervous system in structures like the olfactory bulb or amygdala, first affecting higher central regions such as the amygdala and substantia nigra before descending to the medulla. In both progression models, subcortical limbic structures such as the amygdala, thalamus, hypothalamus, and hippocampus show pathological changes before widespread cortical lesions develop.

The changes observed in the amygdala suggest its importance in “brain-first” type PD. Previous studies have indicated that in brain-first type PD, the amygdala shows pathological changes from early stages, which are associated with early cognitive dysfunction (Borghammer et al., 2022; Horsager and Borghammer, 2024). α-synuclein pathology accumulation in the amygdala may serve as a starting point for pathological expansion to other brain regions, potentially leading to cognitive and emotional dysfunction. Notably, previous research has shown a concentration of α-synuclein pathology in the basolateral nucleus of the amygdala (Harding et al., 2002). In the thalamus, MRI studies have confirmed iron accumulation, suggesting that this abnormality aligns with the distribution of α-synuclein pathology and is closely related to cognitive decline (Thomas et al., 2020). In contrast, DTI studies have shown marked deterioration in the microstructure of projection fibers associated with specific thalamic nuclei, but the fine regions detected in our study have not been evaluated (Planetta et al., 2013). Furthermore, arterial spin labeling studies have reported that decreased blood flow in the dorsomedial nucleus of the thalamus is closely related to cognitive decline (Azamat et al., 2021). Additionally, a significant reduction in overall thalamic volume has been observed in patients with PD, which may be an early phenomenon associated with disease progression (Lee et al., 2011). Functional MRI studies have shown how hypothalamic degeneration is related to autonomic dysfunction in patients with PD (Dayan and Sklerov, 2021). Hypothalamic degeneration may indirectly affect cognitive function through autonomic nervous system and metabolic regulation (Schrag et al., 2017).

However, contrary to our expectations, the results indicated that cerebral cortical regions were more strongly associated with cognitive impairment than the amygdala, thalamus, hypothalamus, and hippocampus. In the comparison of corrected brain volumes between PDCI and PDNC, although a thalamic subregion (left lateral geniculate nucleus) was significantly smaller in PDCI, structural changes were extensively observed across cortical regions including the temporal, parietal, and occipital lobes. Furthermore, our correlation analysis between regions demonstrating significant volumetric differences in PDCI versus PDNC and cognitive function measures identified significant associations limited to the corrected brain volumes of cortical regions. These findings align with previous research on the relationship between specific cortical regions and cognitive function. The left superior temporal gyrus, which showed significant volumetric differences in our study, is associated with a decline in memory and attention/processing speed. MRI studies have shown that decreased cortical thickness in this region is related to reduced memory function (Lee, 2023). Furthermore, magnetoencephalography studies have revealed that decreased functional connectivity between the left superior temporal gyrus and other brain regions is associated with reduced attention and processing speed (Wiesman et al., 2016). Gray matter volume reduction in the right lateral occipital region is associated with physical frailty and cognitive impairment (Chen et al., 2019), while decreased glucose metabolism in this area is closely related to memory function decline (Zhihui et al., 2023). Gray matter reduction and decreased functional connectivity in the right inferior parietal region are associated with a decline in visuospatial and executive functions (Zhang et al., 2018; Kawashima et al., 2021). The left rostral anterior cingulate is linked to decreased attention function, with observed reduced blood flow during attention-demanding sentence processing tasks (Grossman et al., 1992). Degeneration in the left transverse temporal white matter region has been linked to decreased visuospatial function, indicating that white matter degeneration affects cognitive function in patients with PD (Auning et al., 2014).

Hazard ratio analysis was conducted to further investigate the risk of MCI onset associated with structural changes in these regions. While structural changes in the left lateral geniculate nucleus, a thalamic subregion, represented one risk factor, structural changes across multiple cerebral cortical regions were identified as primary risk factors for MCI onset. Multiple regions showed statistically significant hazard ratios, suggesting that cognitive decline in PD is caused by changes in multiple regions and overall network changes, rather than changes in a single brain region (Suo et al., 2022; Pereira et al., 2015). This highlights the complex, interconnected nature of brain changes associated with cognitive decline in PD. It also suggests that a more comprehensive approach is needed to predict the risk of cognitive decline, rather than just focusing on changes in individual brain regions (Caspell-Garcia et al., 2017; Liu et al., 2017; Schrag et al., 2017; Kalaitzakis and Pearce, 2009; Legault-Denis et al., 2021).

The findings in our study that regarding the absence of correlation of volume changes in subcortical structures with cognitive function and the association of changes in cortical regions with cognitive function appear to contradict the progression sequence of alpha-synuclein pathology (subcortical → cortical). One mechanism explaining this contradiction is “propagation along neuronal synaptic connections” (Agosta et al., 2015). Alpha-synuclein pathology may spread not only to anatomically adjacent regions but also between synaptically connected neurons, propagating along anatomically connected neural pathways in a “prion-like” manner (Hijaz and Volpicelli-Daley, 2020). When misfolded alpha-synuclein forms in certain neurons, it can be taken up by adjacent cells through synapses and trigger chain aggregation of endogenous alpha-synuclein (Jucker and Walker, 2013; Guo and Lee, 2014). Through this trans-synaptic propagation, pathology that begins in parts of the brainstem, amygdala, or basal forebrain may spread directly to the cerebral cortex along anatomical neural connections, potentially causing structural abnormalities in the cerebral cortex earlier than expected in the Braak hypothesis. A second mechanism is “dysfunction due to neural network disconnection.” Neural networks involved in cognitive impairment in PD include dopaminergic, cholinergic, and noradrenergic networks (Gratwicke et al., 2015). The cholinergic neural network has been particularly implicated in early cognitive decline in PD (Ray et al., 2018; Crowley et al., 2024). In PD, alpha-synuclein pathology also appears in cholinergic neurons of the basal forebrain, such as the nucleus basalis of Meynert (NBM). Dysfunction of extensive cholinergic projections from the NBM can reduce synaptic input to the cortex, cause deficiency of neurotrophic factors (Ghosh et al., 1994; Mogi et al., 1999), and potentially lead to secondary atrophy of cortical neurons. In our study, the temporal, parietal, and occipital lobes, which showed structural abnormalities related to cognitive dysfunction, are regions incorporated into the cholinergic neural network. Thus, cortical atrophy and cognitive decline may be indirectly caused by degeneration of subcortical projection systems before alpha-synuclein pathology itself accumulates in the cortex, suggesting phenomena that cannot be explained by the simple equation of “cortical degeneration = presence of alpha-synuclein pathology in that region.” Additionally, pathological mechanisms related to Alzheimer’s disease (AD) may be involved. Patients with PD, especially those with dementia, often have comorbid brain pathologies characteristic of AD, such as amyloid-β plaques and neurofibrillary tangles of tau protein (Irwin et al., 2013). However, AD pathology is prominent in the limbic system and medial temporal cortex (Braak et al., 2006). Therefore, the involvement of AD pathology alone cannot explain the mechanism by which structural abnormalities occur in the cortex earlier than in the subcortical regions.

Our research demonstrates that central nervous system degeneration in PD does not necessarily progress unidirectionally toward atrophy and does not always directly correlate with symptoms. The PDCI group showed significantly larger adjusted brain volumes in the right caudal anterior cingulate cortex compared to that in the HC group, and in the left rostral anterior cingulate cortex compared to that in the PDNC group. Furthermore, the PDNC group showed significantly larger adjusted brain volumes in the left transverse temporal gyrus and right inferior parietal lobule compared to that in the HC group. In the correlation analysis between cognitive tests and brain regions, increased adjusted brain volume in the left rostral anterior cingulate cortex (a risk factor for cognitive impairment) and decreased white matter in the left transverse temporal gyrus showed negative correlations with cognitive test scores. This phenomenon suggests that compensatory mechanisms in PD MCI are thought to occur particularly during the early course of the disease (Legault-Denis et al., 2021).

The mechanisms of cortical impairment and cognitive decline in PD involve the interaction of multiple systems, including pathological changes (Kalaitzakis and Pearce, 2009), network-level degeneration (Gratwicke et al., 2015), and compensatory adaptation (Legault-Denis et al., 2021; Appel-Cresswell et al., 2010), among others (Biundo et al., 2016). Notably, this complex interaction suggests that abnormalities in neural circuits are already causing cognitive impairment before α-synuclein pathology clearly progresses to the cortex (Laansma et al., 2021). Therefore, as early diagnosis and intervention are important, interventions targeting specific brain regions or approaches aimed at improving function at the network level could be considered. These may include pharmacotherapy targeting specific neural circuits, non-invasive brain stimulation therapy, or cognitive function training. Future studies should focus on these aspects.

Our study has several limitations. First, we analyzed only data from subjects in the PPMI dataset who underwent imaging according to the specific protocol described in the MRI section. This approach was necessary to minimize variability in imaging conditions when analyzing fine neuroanatomical structures such as the amygdala, thalamus, and hypothalamic subregions. Similar participant selection based on specific MRI protocols has been employed in previous studies using PPMI data. However, we acknowledge that this selective approach may introduce selection bias, as our analysis was restricted to subjects meeting these specific imaging criteria. This limitation potentially affects the generalizability of our findings to the broader PD population.

Second, our selective inclusion of subjects with identical MRI protocols resulted in a limited sample size. The PDCI group, in particular, had a relatively small sample size, which may have affected the statistical power of our analyses. Validation studies with larger sample sizes will be necessary to confirm the findings obtained in this study.

Third, our use of MoCA as the primary criterion for MCI determination warrants discussion. While the MDS Task Force guidelines suggest a more comprehensive Level II diagnostic approach using multiple cognitive domain tests, we employed MoCA (Level I) based on several considerations. MoCA has been validated as an effective tool for assessing cognitive function in patients with PD and has demonstrated reliability in predicting cognitive decline. Studies have shown comparable effectiveness between Level I and Level II criteria in identifying PD-MCI, and MoCA has proven particularly useful for predicting long-term cognitive outcomes. Furthermore, this approach aligns with real-world clinical practice, where time constraints often necessitate the use of screening tools rather than extensive neuropsychological batteries. Nevertheless, we acknowledge that a more comprehensive neuropsychological assessment might have provided deeper insights into domain-specific cognitive impairments. Our baseline data revealed that the PDCI group showed lower performance across several cognitive domains than the PDNC group, suggesting that subtle cognitive changes might be detectable through detailed domain-specific testing. Future research combining both screening tools and comprehensive neuropsychological assessments could better illuminate the relationship between specific cognitive domain impairments and structural brain changes in early PD. Despite these limitations, this study provides important insights into the relationship between early brain structural changes and cognitive decline in PD.

Finally, our study discusses potential neural degeneration mechanisms beyond the conventional model of α-synuclein pathology propagation based on the distribution of brain structural abnormalities in PD with mild cognitive impairment. However, we did not conduct direct analyses using α-synuclein biomarkers. While we examined structural changes in regions where α-synuclein pathology appears relatively early (amygdala, thalamus, and hypothalamus) versus later (cerebral cortical regions), we could not directly confirm the progression of α-synuclein pathology in individual subjects. In the future, it will be important to investigate the correlation between α-synuclein levels and region-specific atrophy using biomarkers such as cerebrospinal fluid (Rossi et al., 2020) and blood (Okuzumi et al., 2023) test data obtained through real-time quaking-induced conversion methods. Despite these limitations, this study provides important insights into the relationship between early brain structural changes and cognitive decline in PD.

In conclusion, this study investigated structural changes in subcortical brain regions preceding cognitive impairment in PD, revealing specific alterations in the amygdala, thalamus, and hypothalamus. These findings suggest that structural abnormalities may appear in the cerebral cortex before the stage proposed by conventional α-synuclein propagation models, potentially involving multiple mechanisms beyond α-synuclein propagation, including global neural circuit dysfunction, disruption of neurotransmitter systems, breakdown of compensatory mechanisms, and coexisting pathologies (beta-amyloid and tau proteins). This insight may serve as a foundation for developing early diagnostic biomarkers and new therapeutic strategies targeting neural circuits and neurotransmitter systems.

## Data Availability

Publicly available datasets were analyzed in this study. This data was acquired on February 23, 2023 from the Parkinson’s Progression Markers Initiative (PPMI) database (https://www.ppmi-info.org/access-data-specimens/download-data), RRID:SCR_006431. PPMI is supported by the Michael J. Fox Foundation for Parkinson’s Research and its funding partners, as listed on the PPMI website. Access to PPMI data requires signing the Data Use Agreement, submitting an Online Application, and adhering to the study’s Publications Policy. Requests for data access should be directed to PPMI via https://ida.loni.usc.edu/collaboration/access/appLicense.jsp.

## Footnotes

^1^ https://www.ppmi-info.org/

^2^ http://surfer.nmr.mgh.harvard.edu/

^3^ https://surfer.nmr.mgh.harvard.edu/fswiki/SubregionSegmentation

^4^ https://surfer.nmr.mgh.harvard.edu/fswiki/ScLimbic

^5^ https://www.python.org
